# Meta-Analysis of the Gut Microbiome: An African American Representation

**DOI:** 10.3390/ijerph22101591

**Published:** 2025-10-20

**Authors:** Anushka KC, Roshan Paudel

**Affiliations:** Department of Computer Science, School of Computer, Mathematical, and Natural Sciences, Morgan State University, Baltimore, MD 21251, USA; ankc1@morgan.edu

**Keywords:** gut, microbiome, African American, meta-analysis, machine learning, ethnicity

## Abstract

The human gut hosts approximately 100 trillion microbes, forming a complex ecosystem critical to the body’s metabolism, nutrition, and immune function. Despite growing research, African Americans remain underrepresented in clinical studies. This study addresses the gap through a comprehensive meta-analysis of gut microbiome datasets. Fecal sample data from amplicon sequencing were analyzed using a bioinformatics pipeline that incorporated DADA2 for sequence processing and Phyloseq for diversity analysis within RStudio (v2024.09.0+375). Statistical approaches, including Wilcoxon tests, Kruskal–Wallis tests, PERMANOVA, and ANCOM-BC, identified significant microbial differences. Results revealed that African Americans exhibited lower microbial diversity. Beta diversity metrics demonstrated a stronger effect of ethnicity compared to diet, age, sex, and BMI, highlighting its significance in microbiome variation. Similarly, ANCOM-BC identified *Clostridium sensu stricto 1* significantly enriched in healthy African Americans, while *Dialister* was depleted, a finding with potential clinical relevance given previous research linking reduced *Dialister* abundance with depression. Additionally, machine learning approaches were found to potentially complement traditional statistical methods by handling class imbalance and identifying complex microbial associations. By addressing critical gaps in microbiome research, this study underscores the importance of inclusive datasets in enhancing disease risk prediction and ensuring that microbiome-based health interventions are equitable and broadly applicable.

## 1. Introduction

### 1.1. The Global Microbiome

The microbiome is a community of microorganisms that interacts with the host and the environment, contributing to the ecosystem in unique ways [1]. For example, bacteria residing in the gut can aid in digestion, synthesize important nutrients such as vitamin K, and even promote angiogenesis [2]. In soil, microorganisms facilitate development and plant growth through symbiotic relationships by cycling carbon, nitrogen, and other elements [3]. Similarly, it is believed that the first synthesizer of oxygen was cyanobacteria, and even today, microorganisms in the oceans make up half of the oxygen on Earth [4]. The dynamic interplay between microbiomes, their hosts, and surrounding environments underscores their fundamental importance in sustaining the natural ecosystem and promoting health across diverse biological systems.

### 1.2. The Gut Microbiome

Considering the microbiome in different environments, one of the most important environments related to human health is the gut microbiome. The human gut microbiome is a dynamic and complex community of microorganisms that includes bacteria, fungi, viruses, and archaea residing in the gastrointestinal tract. These microbes play a fundamental role in digestion, metabolism, immune regulation, and even neurological function. With about 100 trillion microbes, human cells are at least outnumbered by a factor of one—the gut microbiome is now regarded as an essential organ-like entity [5]. Its influence goes beyond gut health, with new evidence linking microbiome composition to metabolic disorders, inflammatory diseases, mental health issues, and cancer development [6].

Given its extensive genetic and functional diversity, the gut microbiome offers a remarkable opportunity for scientific discovery [7]. Researchers are now studying how microbial imbalances contribute to disease and how microbiome-targeted therapies could enhance health outcomes. However, gaps still exist in our understanding, especially regarding microbiome differences across various populations.

Although the gut microbiome has gained significant attention in modern science, its recognition goes back much earlier. In the 17th century, Antonie van Leeuwenhoek (1632–1723) was the first to observe and document microorganisms from stool and saliva samples [8]. Even before that, ancient medical traditions recognized the gut’s importance. Hippocrates famously said that “all disease begins in the gut,” a view also echoed in Ayurveda, the traditional Indian medicine system (dating back to 1500 BCE). Ayurvedic practices, such as emphasizing dietary diversity, fermented foods, fiber-rich plants, and herbs, are now understood to help support a healthy gut microbiome [9].

Despite these early insights, it was not until the 19th century that scientists began to realize the importance of intestinal microbes. The development of germ theory, probiotics, and the discovery of the gut’s extensive neural networks, sometimes called the “second brain,” laid the groundwork for modern microbiome research [8].

To deepen our understanding of the gut microbiome’s role in health and disease, researchers increasingly use meta-analysis, a statistical approach that combines findings from multiple studies. This method allows for cross-validation of associations across different populations and study designs, leading to new discoveries that individual studies might miss due to limited sample sizes or specific demographic constraints. For example, essential microbial and metabolic biomarkers linked to colorectal cancer have been identified through a meta-analysis, revealing patterns that can support early diagnosis and intervention processes [10].

However, microbiome research remains disproportionately focused on certain populations, leaving substantial gaps in knowledge about underrepresented groups. For example, in a 2018 study on gut microbiome diversity across ethnicities in the United States, only 13 African American (AA) participants out of 1375 individuals were included, highlighting a significant imbalance in research representation [11]. To address this disparity, this study systematically compiled and analyzed microbiome datasets, with a focus on African Americans. By combining findings from various studies, this research seeks to promote a more inclusive and comprehensive understanding of the gut microbiome’s role in health and disease.

## 2. Materials and Methods

### 2.1. Study Population and Data Collection

A literature review and search initially identified studies that conducted metagenomic and metabolomic analyses of gut microbiome data, focusing on African Americans or other minority groups. The next step was to select studies based on the inclusion criteria: (a) studies used fecal samples, (b) employed 16S ribosomal RNA (rRNA) amplicon sequencing, (c) metadata included demographic information, and (d) raw sequence data were available for download. Studies were excluded if they lacked demographic data or involved populations of infants and pregnant women. When raw sequence data or metadata were not publicly available, the authors of the papers were contacted via email to request the data.

Initially, the Microbial Metabolites Database (MiMeDB) and the Microbiome Metabolome Integration Platform (MMIP) were used to search for microbiome and metabolome data. However, due to the lack of straightforward demographics data, Google Scholar and PubMed were used along with Gene Expression Omnibus (GEO). “gut microbiome” AND “metabolome” AND ““16S” OR “Amplicon”” AND “fecal” was the search query used for conducting a general search and to gain an understanding of the research present so far in this field. Then, a more focused search using the query “gut” AND “microbiome” AND “metabolome” AND “16s rRNA” OR “amplicon” AND “African American” AND “PRJ” OR “PRE” was conducted. The new query with the added variables was necessary to gain a clear understanding of whether research has covered African Americans. The “PRJ” and “PRE” queries were added to ensure the papers included data that could be accessed through the National Center for Biotechnology Information (NCBI) or the European Molecular Biology Laboratory’s European Bioinformatics Institute (EMBL-EBI). 571 results were retrieved from Google Scholar. PubMed resulted in only fourteen results (PRJ and PRE queries had to be removed to find relevant papers since adding those resulted in zero results).

**Figure 1:**
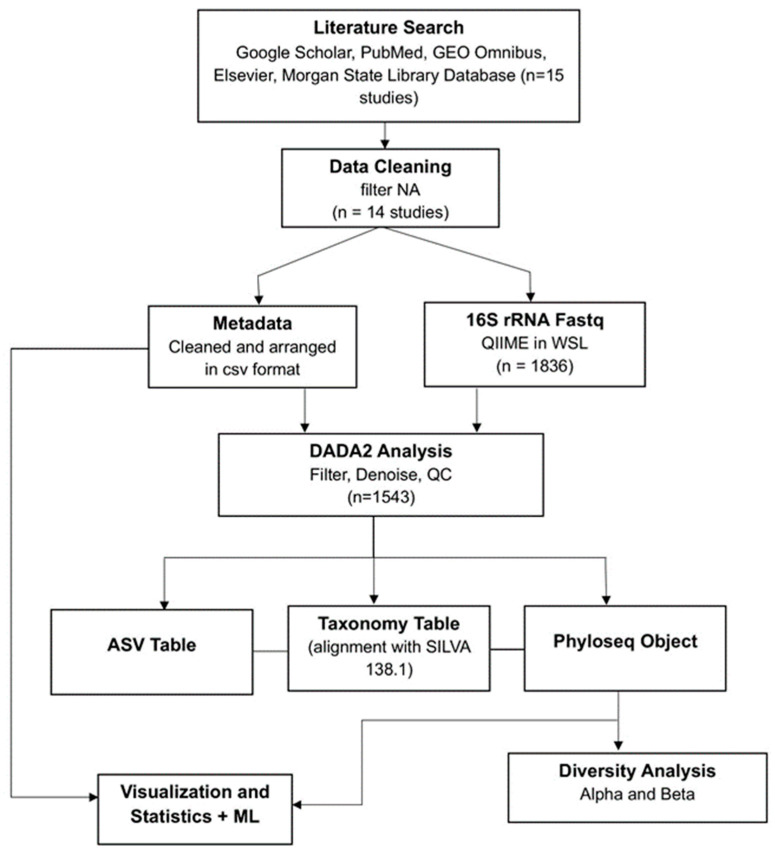
Methodology Flowchart: Raw 16S rRNA sequencing data were collected, followed by data cleaning, metadata organization, and fastq files processing using QIIME2 and DADA2 analysis. Outputs included amplicon sequence variant (ASV) tables, taxonomy tables (aligned against the SILVA 138.1 reference database), and Phyloseq objects. These datasets were used for downstream analyses, including diversity assessment (alpha and beta diversity), visualization, statistical testing, and machine learning applications.

The search resulted in identifying fifteen studies containing 16S rRNA fecal microbiome data. One study was excluded because demographic data were requested from the authors but not available, leaving a final total of fourteen studies. The National Center for Biotechnology Information Sequence Read Archive (NCBI SRA) public repository was the source from which raw FASTQ files were downloaded. Metadata was collected either from the supplementary information of the study or from SRA. Samples from individuals under eighteen and pregnant women were not included in the analysis. The methodology flowchart of the entire process is shown in Figure 1.

For the purposes of this study, ethnic groups were classified as follows: African Americans (U.S. citizens of African descent, distinguished from the broader Black category), Caucasian (individuals of European descent worldwide), Asian (East, South, and Southeast Asian descent), Hispanic/Latino (individuals of Latin American descent), Native American (indigenous peoples of North America), Multiracial (individuals identifying with two or more racial categories), and Other (individuals not fitting the above categories). See Tables S1 and S2 in the Supplementary.

### 2.2. Data Processing and Quality Control

Raw FASTQ files were downloaded and split into their respective forward and reverse files using QIIME. Folders for each study were created chronologically from 1 to 14, and related files were saved in their respective folders. The sequence data for each study were filtered to a maximum of 10,000 reads. DADA2 in RStudio was used to process raw fastq files and obtain the ASV tables.

#### 2.2.1. Metadata Coercion

To ensure consistency in the metadata across studies, column names were systematically standardized. First, relevant columns such as Run, BioProject, Age, Sex, BMI, Country, Continent, Ethnicity or Race, and Treatment or Condition were extracted and uniformly formatted. While demographic data were included whenever possible, for studies conducted in specific countries, geographic proxies were used to infer ethnicity—for example, samples from India and China were classified as “Asian” (n = 175), and samples from an Italian cohort (n = 31) were classified as “Caucasian” [12]. All other studies contained explicit demographic data. This approach aimed to include data from Asian populations not covered in previous meta-analyses. When Age and BMI data were unavailable for individual samples, mean values reported in the papers were used and added to the metadata. The Treatment/Condition column was replaced with a new “Study.Group” column, indicating whether patients were “Healthy” or “Diseased,” with careful verification that treatment samples were collected before intervention. Table S2 shows the breakdown of the “Healthy” versus “Diseased” samples for each study.

#### 2.2.2. DADA2

DADA2 is an RStudio package designed for modeling and correcting errors in Illumina-sequenced amplicon data. It accurately infers sample sequences, differentiating sequences by as little as one nucleotide without grouping them into Operational Taxonomic Units or OTUs [13]. Instead, a quality-aware error model enables the construction of Amplicon Sequence Variants (ASVs). DADA2 enhances the accuracy of diversity and dissimilarity measurements, potentially allowing for strain-level variation studies using amplicon methods. Unlike methods that create OTUs, DADA2 preserves biological information by precisely reconstructing amplicon-sequenced communities at high resolution [13]. In this study, the DADA2 pipeline was used for quality filtering, denoising, and merging amplicon sequence data from the downloaded 16S rRNA sequencing data [13]. All procedures were conducted in R version 4.0.3, with the DADA2 pipeline implemented via the dada2 package (v1.16.0). Additional visualizations were carried out using the ggplot2 (v3.3.3) and phyloseq (v1.32.0) packages. Taxonomic assignment was performed by comparing sequences against a SILVA database (silva_nr99_v138.1_train_set.fa) as the reference.

#### 2.2.3. Statistical Analysis

Data was analyzed using specific groupings for comparison: (a) Grouping by Health Status–Healthy versus Diseased, (b) Grouping by African American (AA) Status–AA versus Non-African Americans (non-AA), and (c) Grouping by Health Status within AA–Healthy AA versus Healthy non-AA.

Alpha diversity is a measure of within-sample diversity [14]. This measure encompasses the number of different organisms, also referred to as richness, and the evenness of their distribution [14]. The two commonly used indices for alpha diversity are the Shannon Index and the Simpson Index [15,16]. While the Shannon index is useful for assessing the overall community structure, the Simpson Index, with its focus on more dominant species, can highlight if certain taxa are overrepresented within a sample.

Alpha diversity was calculated using unfiltered counts from the Phyloseq object. Shannon and Simpson indices were computed, and their significance was assessed with the Wilcoxon Rank Sum Test and Kruskal–Wallis Test. *p*-values from pairwise comparisons were adjusted with the Benjamini–Hochberg (BH) procedure to control the false discovery rate [17]. This adjustment was necessary to reduce false positives when performing multiple statistical tests across different demographic groups.

Beta diversity measures the between-sample diversity, revealing how species composition varies across different locations or communities [14]. It enables a spatial understanding of biodiversity patterns by quantifying the differences between groups. One method of this spatial approach is through Bray–Curtis dissimilarity, a statistical measure that assesses the dissimilarity in species composition between two sites based on the abundance data of the gut microbiome [18]. In this study, this measurement was applied to compare microbial communities between ethnicities.

Since data varied in the number of total reads, normalization was conducted by Cumulative Sum Scaling (CSS), where all samples were scaled to the sequencing depth of the smallest sample [19]. This normalization approach was conducted to mitigate bias from samples with a higher number of reads. Finally, Permutational Multivariate Analysis of Variance (PERMANOVA), a non-parametric test that assesses whether the centroids and dispersion of groups differ in multivariate space, was conducted to determine whether observed differences between ethnic groups were statistically significant [20].

The merged phyloseq object created from the DADA2 analysis was used to develop a taxonomic tree for a straightforward visualization of microbial composition of all studies. The treeio package and ggtree package were used for the tree construction and visualization, respectively [21,22]. A prevalence analysis of the complete dataset was also conducted by using the abundance data of the top genera in each ethnic group to examine how they differ from one another.

Observing the Firmicutes/Bacteroidota ratio is a common method for understanding the composition of bacteria in samples, as they consistently dominate the gut microbiome. After aggregating taxa to the phylum level, the total count per sample for each phylum was obtained. Then, the Log_2_ ratio of Firmicutes over Bacteroidota was calculated. The mean of the F/B ratio was also calculated to get a better visualization between healthy and diseased African Americans versus other ethnicities.

Differential abundance analysis (DAA) is another important statistical measure for identifying microbial taxa that differ significantly in abundance between conditions or groups. One method of DAA is the Analysis of Compositions of Microbiomes with Bias Correction (ANCOM-BC) [23]. ANCOM-BC corrects for sample-specific biases that can affect microbiome data, allowing researchers to identify which specific bacteria are truly different between groups by analyzing absolute abundance rather than just relative proportions [23]. This analysis was conducted to investigate the differential abundance of microbial composition in the complete dataset between healthy AA and healthy non-AA.

#### 2.2.4. Preliminary Machine Learning Application for Ethnicity Prediction

Although Random Forest is one of the methods consistently demonstrating optimal performance in microbiome data prediction, this meta-analysis aims to explore newer algorithmic approaches [24]. Using the extensive feature set available, gradient boosting algorithms were chosen to assess potential improvements in predictive modeling beyond traditional Random Forest methods, such as LightGBM, XGBoost, and AdaBoost. Random Forest was also used for comparison.

Gradient boosting algorithms are machine learning methods that build decision trees sequentially, with each new tree aiming to correct the errors of the combined previous trees, thereby gradually enhancing the overall prediction accuracy [25]. AdaBoost, introduced in 1995, was a pioneer in this area, emphasizing learning from weaker models [26]. XGBoost further advanced the field by adding regularization and system optimization, which boosted both prediction accuracy and efficiency [27]. The latest algorithm extends these concepts using innovative sampling and feature compression techniques [28].

The ASV and taxonomy tables from the merged phyloseq object were exported as individual .csv files. For the ASV data, normalization was performed using log transformation. The exported data files were then used for machine learning (ML) analysis in Google Colab.

Google Colaboratory (Colab) is a cloud platform that allows free, browser-based execution of Python (v 3.11) code. It has become popular for machine learning development because of its accessible GPU resources and collaborative features [29]. The exported CSV files—ASV, taxonomy, and metadata—were combined into a single data frame called merge_all and saved as a CSV file.

Missing values in the metadata were addressed using suitable domain strategies: numerical features were filled with zeros, and categorical features were replaced with empty strings. Features with few occurrences (n < 6) were eliminated to decrease dimensionality and reduce noise.

The data was divided into train (80%), validation (10%), and test (10%) sets. These splits were stratified to keep class distributions consistent across all partitions, ensuring that each subset accurately represents the original data. The preprocessor was designed to identify categorical and numerical features, with categorical features being transformed using one-hot encoding. After preprocessing, hyperparameter tuning was performed for each candidate model to mitigate overfitting in the machine learning models by reducing model complexity and controlling learning behavior. For LightGBM, tree depth was limited (max_depth = 5), a moderate learning rate was used (learning_rate = 0.1), and the number of trees was restricted (n_estimators = 100). Similarly, XGBoost employed max_depth = 5, learning_rate = 0.1, and n_estimators = 100 to prevent overly complex trees while maintaining learning stability. For AdaBoost, the default base estimator is a shallow decision tree (stump), and the learning rate was reduced to 0.1 to limit the contribution of each weak learner. Random Forest leverages inherent bagging and feature randomness, with a moderate number of trees (n_estimators = 100) and class_weight = ‘balanced’ to prevent bias toward majority classes. Collectively, these hyperparameter choices were designed to reduce overfitting while preserving predictive performance across all models.

Initially, models were trained on the training set and then iteratively refined based on validation set performance metrics. The final evaluation used the test set to unbiasedly assess the predictive performance of all the algorithms implemented.

## 3. Results

From the data collection, a total of 1836 amplicon sequenced samples were considered for the data analysis consisting of different ethnicities: African American (n = 274), Asian (n = 307), Caucasian (n = 1028), Hispanic/Latino (n = 160), Native American (n = 3), Multiracial (n = 55), and Other (n = 9). After DADA2 analysis, which included filtering and quality control, the remaining samples were: African American (n = 245), Asian (n = 250), Caucasian (n = 925), Hispanic/Latino (n = 85), Native American (n = 2), Multiracial (n = 27), and Other (n = 9). Native American, Multiracial and Other were combined into Multiracial/Other for analysis since the sample sizes were small.

### 3.1. Alpha Diversity

**Figure 2:**
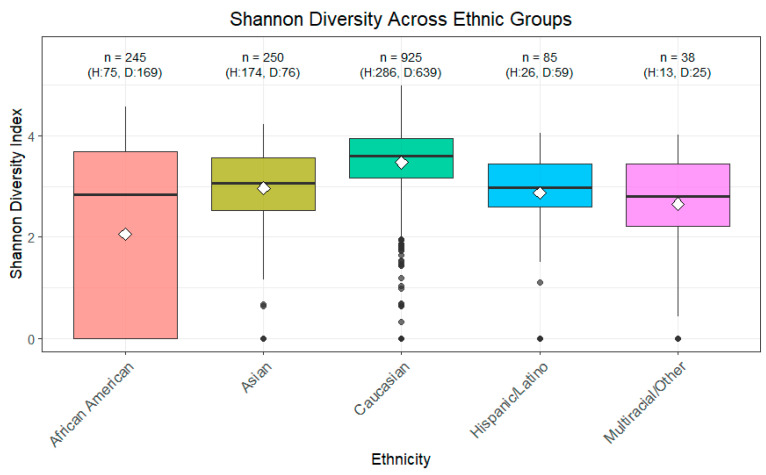
Shannon Diversity across Ethnic Groups. Shannon diversity index, which measures both richness and evenness of microbial taxa, was calculated for each sample. Higher values indicate greater microbial diversity. Boxplots represent the median, interquartile range, and outliers for each ethnic group. Statistical significance between groups was assessed using Wilcoxon and Kruskal–Wallis tests. This analysis highlights potential differences in microbiome diversity associated with ethnicity.

The Shannon Diversity Index across various ethnicities, analyzed using pairwise Wilcoxon tests with BH adjustment, showed significant differences (*p* < 0.05) between all groups (Figure 2). Additionally, a Kruskal–Wallis test considering the different levels confirmed these differences as highly significant (*p* < 0.05).

The samples were grouped into healthy and diseased categories to examine differences in diversity. The Wilcoxon test showed significance as well (*p* < 0.05). Grouping AA and non-AA also showed significant differences with the Wilcoxon test (*p* < 0.05), with AA showing lower diversity.

**Figure 3:**
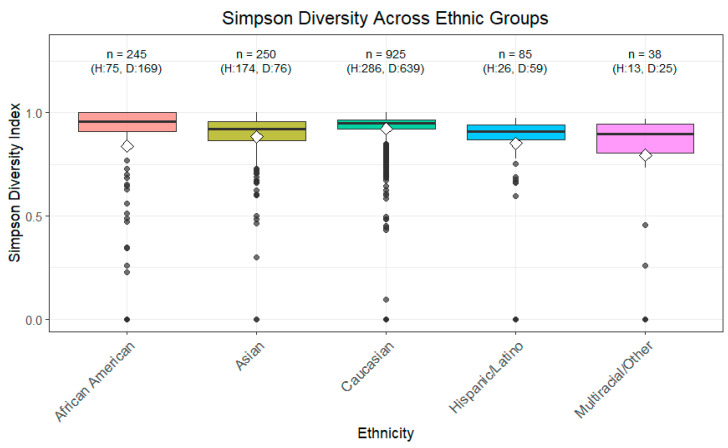
Simpson Diversity across Ethnic Groups. The Simpson diversity index, which accounts for both richness and evenness of microbial taxa with greater emphasis on dominant species, was calculated for each sample. Higher values indicate higher diversity, with lower values reflecting dominance by a few taxa. Boxplots show the median, interquartile range, and outliers for each ethnic group. Statistical differences between groups were evaluated using Wilcoxon and Kruskal–Wallis tests. This visualization illustrates potential variations in microbial community structure associated with ethnicity.

Simpson diversity also showed significant differences for ethnicity, as determined by the Kruskal–Wallis test, as well as pairwise comparisons using the Wilcoxon test (Figure 3). Health status barely reached significance (*p* = 0.05959). However, Simpson diversity for healthy AA and healthy non-AA showed a significant difference with a Wilcoxon test *p*-value of 0.000288. At the same time, while the mean was lower for AA compared to non-AA, the higher standard deviation points to greater heterogeneity within the AA population.

### 3.2. Beta Diversity

**Figure 4:**
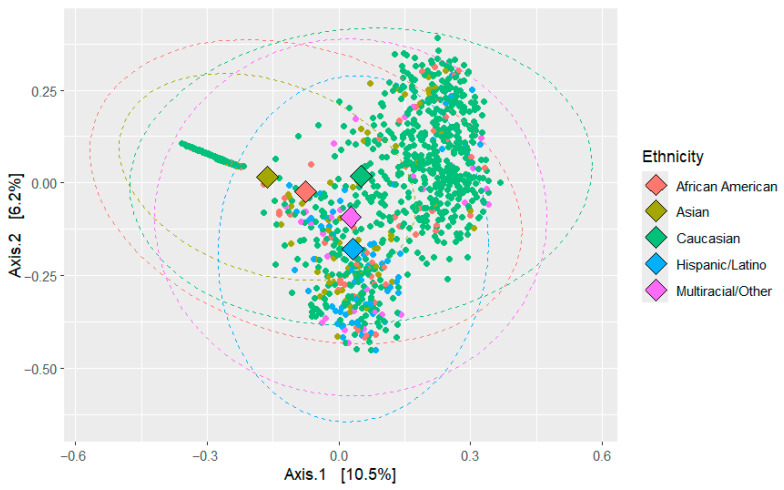
PCoA Analysis of Microbial Composition based on Ethnicity. PCoA was performed based on Bray–Curtis dissimilarity to visualize differences in microbial community composition between samples. Each point represents an individual sample, colored according to ethnic group. Clustering patterns indicate similarity of microbial profiles within and between groups. Axes show the principal coordinates explaining the greatest variation in the dataset. Statistical significance of group separation was assessed using PERMANOVA. This analysis highlights potential differences in overall microbial community structure associated with ethnicity.

Beta diversity was analyzed across different ethnicities, comparing AA and non-AA, as well as healthy and diseased groups. The analysis used Principal Coordinate Analysis (PCoA) based on Bray–Curtis dissimilarity, with PERMANOVA conducted using 9999 permutations (see Figure 4). This permutational multivariate analysis of variance evaluated how various factors influenced microbial community composition, reaffirmed with 9999 permutations.

The PERMANOVA results indicated that ethnicity was significantly associated with microbial composition (R^2^ = 0.0346, F = 7.952, *p* < 0.0001). Health status also had a significant effect (R^2^ = 0.00502, F = 6.745, *p* < 0.0001), as did AA status (R^2^ = 0.0038, F = 5.1035, *p* < 0.0001), sex (R^2^ = 0.00374, F = 5.0254, *p* < 0.0001), age (R^2^ = 0.01663, F = 22.607, *p* < 0.0001), and BMI (R^2^ = 0.00446, F = 5.9879, *p* < 0.0001). These findings suggest that microbial community composition is driven by a combination of all these factors.

To assess the homogeneity of dispersion among groups, a permutation test for homogeneity of multivariate dispersion was performed. The test revealed significant differences in dispersion for ethnicity (F = 32.537, *p* < 0.0001), health status (F = 31.921, *p* < 0.0001), age (F = 5.761, *p* < 0.0001), and BMI (F = 7.0923, *p* < 0.0001). However, differences in dispersion for AA status (F = 2.5386, *p* = 0.1108) and sex (F = 2.211, *p* = 0.1344) were not significant.

These results indicate that while all tested variables significantly influenced microbial community composition, some factors (such as ethnicity and health status) also showed significant variation in dispersion, suggesting differences in variability among groups. This is further strengthened by the Principal Coordinate Analysis (PCoA) of Bray–Curtis dissimilarities, which revealed clustering patterns based on both ethnicity and health status. The first two axes explained 16.7% of the total variation (Axis 1: 10.5%, Axis 2: 6.2%).

### 3.3. Microbial Composition

**Figure 5:**
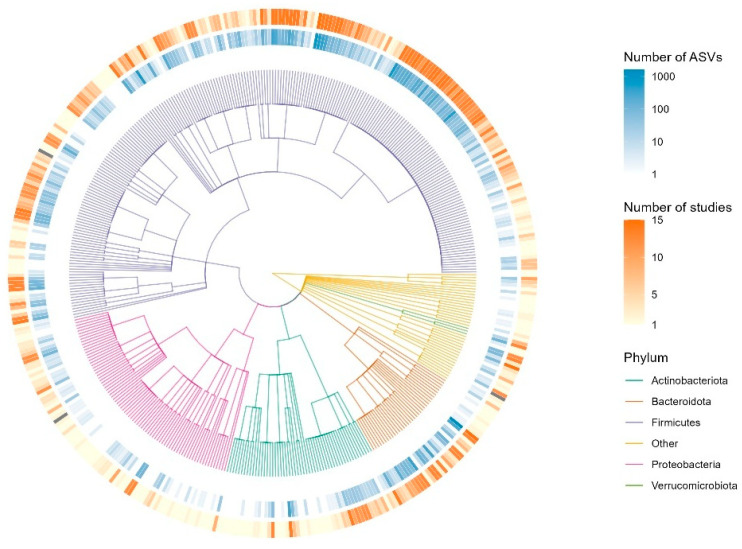
Taxonomic Tree of Microbial Composition for Complete Dataset. The circular taxonomic tree displays phylogenetic relationships among microbial taxa identified in the dataset. Branches are colored according to phylum (Actinobacteriota, Bacteroidota, Firmicutes, Proteobacteria, Verrucomicrobiota, and others). The inner ring indicates the number of amplicon sequence variants (ASVs) per taxon (blue gradient), while the outer ring represents the number of studies in which each taxon was reported (orange gradient). This visualization highlights both taxonomic diversity and study representation across major microbial phyla. The tree construction approach was adopted from a previously published method.

A taxonomic tree was constructed by adopting the workflow from a previously published method [30]. It presents the top 5 phyla across the 15 studies and gives a quick overview of the composition of microbiota (Figure 5). Looking further into the composition required calculating relative abundances. Firmicutes (51.23%) were the most abundant bacteria, followed by Bacteroidota (36.11%), Proteobacteria (4.04%), Actinobacteria (3.86%), Verrucomicrobiota (2.21%), and Euryarchaeota (1.77%). The remaining taxa constituted 0.78% of the total composition.

The analysis revealed distinct patterns in microbiome composition across ethnic groups. Firmicutes abundance was highest in Caucasian (Mean CLR: 0.449) and Asian (Mean CLR: 0.398) populations, while showing lower levels in Multiracial and Native American groups. Bacteroidota demonstrated peak abundance in Multiracial (Mean CLR: 0.518) and Hispanic/Latino (Mean CLR: 0.417) populations. Notably, the study’s sample sizes varied considerably, with Caucasian participants representing the largest group (n = 925) and Native Americans the smallest (n = 2), which limits statistical inference for the latter group. Verrucomicrobiota and Actinobacteriota remained consistently low across all ethnicities. Microbiome composition variability was most pronounced in Native American and Multiracial groups, suggesting potential genetic or environmental factors influencing bacterial community structures. Center Log-Ratio (CLR) transformation was employed to handle compositional data, providing a normalized view of bacterial abundance across different ethnic groups.

Further prevalence analysis revealed statistically significant differences among specific ethnic groups. The Wilcoxon pairwise comparisons showed: Caucasian vs. African American for Firmicutes (*p* = 9.187050 × 10^−18^), Bacteroidota (*p* = 2.053122 × 10^−8^), and Actinobacteriota (*p* = 2.426465 × 10^−7^); Asian vs. Caucasian for Proteobacteria (*p* = 7.817304 × 10^−7^) and Verrucomicrobiota (*p* = 6.707755 × 10^−27^). Caucasians (shaded in gray in Table 1) consistently showed the highest prevalence across most phyla, with Firmicutes at 99.02%, Bacteroidota at 96.31%, Actinobacteriota at 92.40%, Proteobacteria at 88.93% and Verrucomicrobiota at 62%. Verrucomicrobiota demonstrated the most substantial inter-ethnic variation, from 24.10% in Asians to 43.75% in African Americans and 62% in Caucasians. Multiracial individuals and other groups were excluded from the analysis because their results did not reach significance and their sample sizes were relatively small.

Since Firmicutes and Bacteroidota were the predominant phyla observed, their ratio was also examined. Previous studies have linked the Firmicutes-to-Bacteroidota (F:B) ratio to obesity and its potential role as a disease biomarker. However, its reliability remains debated, as some studies suggest that the correlation is inconsistent, given the substantial variability in F:B ratios even among individuals within the same population due to various influencing factors [31]. Nevertheless, given the high abundance of these two phyla, omitting an analysis of their ratio would overlook potentially valuable insights. This analysis aimed to further explore such variability across different ethnic groups.

**Figure 6:**
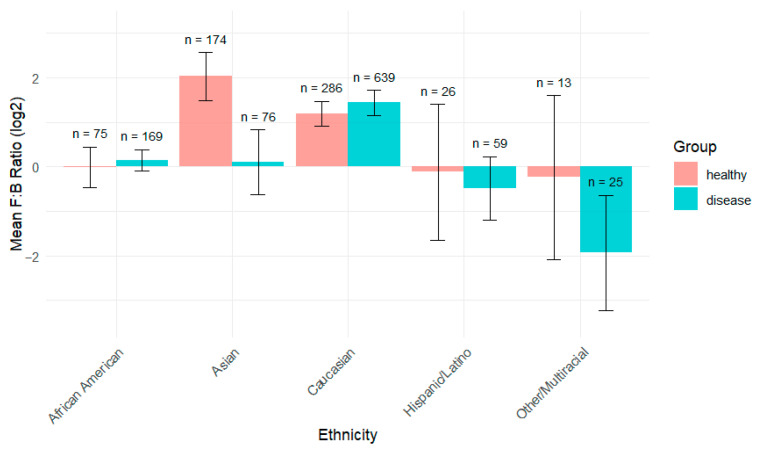
F:B Ratio Comparison by Ethnicity and Disease Status. The mean log_2_-transformed F:B ratio was calculated for each ethnic group and stratified by health status (healthy vs. disease). Bars represent mean values, and error bars indicate the standard error of the mean. Sample sizes (n) for each group are shown above the bars. This visualization highlights differences in the F:B ratio across ethnicities and between health conditions.

Analysis of the Firmicutes-to-Bacteroidetes (F:B) ratio across ethnic groups and disease status in our comprehensive dataset revealed interesting. The F:B ratio is presented on a log_2_ scale, with positive values indicating Firmicutes dominance and negative values indicating Bacteroidetes dominance. Asian populations exhibited the highest F:B ratio in healthy individuals, around 2.0, while Caucasian populations showed relatively high F:B ratios in both groups (Figure 6).

Other/Multiracial and Hispanic/Latino groups show the lowest F:B ratios, close to zero or negative, suggesting a higher prevalence of Bacteroidota compared to Firmicutes. However, these results might be influenced by a small sample size in these groups. In most ethnicities, the healthy (blue) group has a higher F:B ratio than the diseased (red) group. This difference is especially noticeable in Asian and Other/Multiracial populations.

The exception is Caucasians, where those with the disease show a slightly higher F:B ratio. Error bars (black lines) indicate substantial variation within each group. Other/Multiracial shows the highest variability, particularly in the disease group. Asian healthy individuals exhibit considerable variability, despite having the highest mean value.

Different ethnic backgrounds appear to have distinct baseline F:B ratios. Disease states appear to alter this ratio differently across different ethnicities. This suggests that ethnicity should be considered when using the F:B ratio as a biomarker. F:B ratio differences reached significance (*p* < 0.05) between ethnicities for both healthy and diseased individuals via the Wilcoxon test, although the sample sizes compared for Native American and Other/Multiracial groups are very small for each group. This data indicates that microbiome composition varies significantly by ethnicity and health status, suggesting that “normal” or “healthy” microbiome profiles may be ethnicity-specific, and interventions targeting the microbiome might need to be tailored accordingly. F:B ratio differences reached significance (*p* < 0.05) between the ethnicities for both healthy and disease via the Wilcoxon test, although sample sizes compared for Native American and Other/Multiracial is very small for each group.

### 3.4. ANCOM-BC

**Figure 7:**
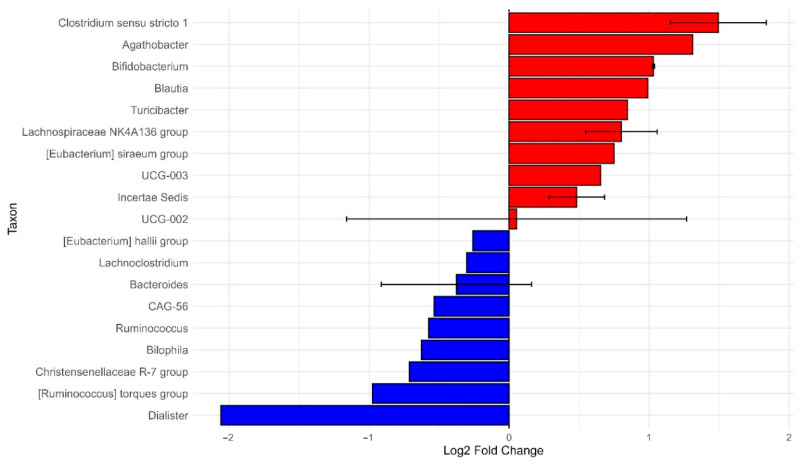
Differentially abundant taxa in African Americans (AA) relative to non-African Americans (non-AA). Bar plot showing significant taxa with log2 fold change in relative abundance. Positive values (red bars) indicate taxa enriched in AA, whereas negative values (blue bars) represent taxa enriched in Non-AA. Error bars denote the standard error of the mean. This analysis highlights key microbial taxa contributing to differences in community composition between groups.

The ANCOM-BC analysis was conducted on Healthy AA versus Healthy non-AA. This was to gain a better understanding of the differences in microbial composition when not confounded by disease status. The study identified 10 statistically significant taxa out of 31 analyzed, with notable variations in bacterial abundance across different general genres (Figure 7).

Genera showed substantial changes in their relative abundance. Dialister stood out as the most depleted taxon in the healthy AA group, with a log_2_ fold change of −2.056 and an extremely low *p*-value of 4.43 × 10^−41^. Conversely, *Clostridium sensu stricto 1* emerged as the most enriched genus, with a log_2_ fold change of +1.848 and a similarly significant *p*-value of 2.35 × 10^−21^.

Most notable changes occurred within the Firmicutes phylum, especially in families such as Lachnospiraceae and Clostridiaceae. Although many taxa showed little variation, the genera with significant changes likely reflect meaningful shifts in microbiome composition. The volcano plot visually supported these results, with blue points indicating taxa that were depleted and red points showing taxa that were enriched in the healthy AA group.

These findings suggest potential ethnic-specific variations in microbiome composition, which may be influenced by genetic, dietary, or environmental factors. The extreme statistical significance of these variations warrants further investigation to understand their potential clinical or biological implications.

### 3.5. Machine Learning for Ethnicity Prediction

To address the issues of confounding variables and the multidimensional nature of microbial data, machine learning was employed to investigate whether ethnicity could be predicted based on the data. Four models were implemented, including LightGBM, XGBoost, AdaBoost, and Random Forest.

**Figure 8:**
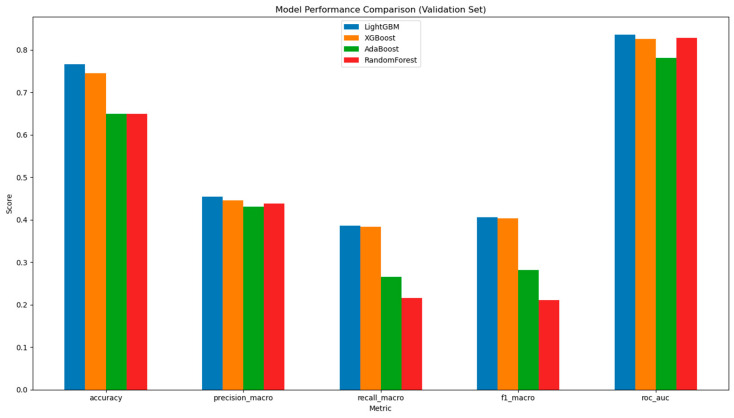
Model Performance Comparison in Validation Set. Comparison is between LightGBM, XGBoost, AdaBoost, and RandomForest for the validation set across multiple evaluation metrics. The *x*-axis represents the five key performance metrics: Accuracy, Precision (Macro), Recall (Macro), F1 Score (Macro), and AUROC. The *y*-axis displays the corresponding scores, allowing for a comparison of each model’s effectiveness.

After training and validation steps, LightGBM performed the best in ethnicity prediction, followed by XGBoost, AdaBoost, and Random Forest. LightGBM and XGBoost performed with similar accuracy (Figure 8 and Table S3).

**Figure 9:**
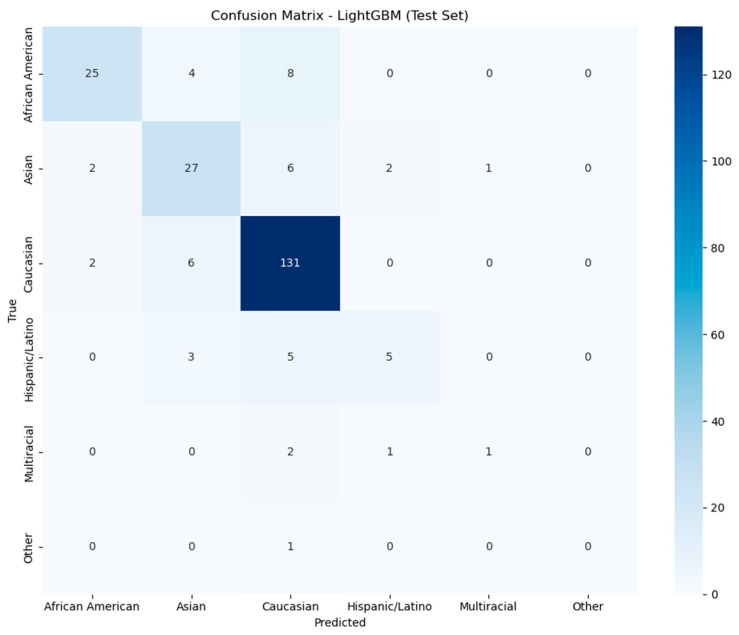
Confusion Matrix visualization from LightGBM model on test set. The *x*-axis represents predicted ethnicity, and the *y*-axis represents the true ethnicity. The intensity of the color scale on the right corresponds to the number of instances the model could predict correctly or incorrectly. The model performed best for Caucasians (131 correct predictions).

The LightGBM model showed variable performance across different ethnic groups. The confusion matrix reveals several important patterns (Figure 9). Caucasians demonstrated the highest prediction accuracy with 131 correct classifications. However, 8 African Americans, 6 Asians, 5 Hispanic/Latino individuals, 2 Multiracial individuals, and 1 individual of another race were incorrectly classified as Caucasian. African Americans achieved moderate prediction accuracy with 25 correct classifications, while 4 were misclassified as Asian and 8 as Caucasian. Asians demonstrated good prediction accuracy, with 27 correct classifications, although some misclassifications were observed across other groups. The Hispanic/Latino group demonstrated poor prediction performance, with only 5 correct classifications out of 13 total individuals. The Multiracial group had very low prediction accuracy with only 1 correct classification out of 4 individuals. Finally, the Other group had no correct classifications for this category. Analysis of feature importance in the LightGBM model revealed several bacterial taxa as meaningful predictors of ethnicity from gut microbiome data (Table 2 and Figure S1). The most influential microbial feature was *Lachnoclostridium* (family Lachnospiraceae), a genus within the Firmicutes phylum, suggesting this bacteria plays a role in carbohydrate and protein metabolism [32].

The next most important bacterial predictors included four distinct *Bacteroides* genera, suggesting they play a significant role in ethnicity-associated microbiome patterns (Table 2). Additional contributors included the *Blautia* genus, also from the Lachnospiraceae family, which are important butyrate producers. Other significant predictors represented diverse taxonomic genera: *Alistipes* (Rikenellaceae), members of the Ruminococcaceae family, *UBA1819* and *Subdoligranulum*, respectively, and an uncultured bacterium from the RF39 order. This taxonomic distribution highlights the importance of Bacteroidetes and Firmicutes phyla, particularly members of the Bacteroidaceae, Lachnospiraceae, and Ruminococcaceae families, in distinguishing gut microbiome compositions between ethnic groups.

## 4. Discussion

In this study, the gut microbiome composition of African Americans was compared with that of other ethnicities to gain a better understanding of this population’s microbial profile. This investigation is the first to do a large-scale data collection and analysis focusing on the African American population. Despite the growing understanding of the gut microbiome’s crucial role in human health, large-scale initiatives have systematically underrepresented African Americans, creating a significant knowledge deficit that potentially impacts health equity and personalized medical interventions.

Our analysis of the African American gut microbiome using the complete dataset uncovered notable taxonomic shifts with possible implications for host health. Alpha diversity analysis showed lower Shannon and Simpson indices in African Americans compared to non-African Americans. The Shannon index measures microbial richness and evenness, indicating that the African American gut community is dominated by fewer species with a less balanced distribution of abundant and rare organisms. The Simpson index, which is more sensitive to dominant species, can suggest gut dysbiosis and increased disease susceptibility [33]. This is concerning, given that African Americans already face greater health disparities than their Caucasian counterparts. Additionally, beta-diversity analyses revealed significant ethnic differences in microbial composition. PERMANOVA demonstrated that ethnicity was significantly associated with microbiome variation, even when controlling for variables like sex, age, BMI, and health status. This indicates that ethnicity should be an important factor in microbiome studies and could serve as a useful comparison metric. Binary testing via PERMANOVA confirmed that being African American or not has a significant impact. Consequently, ethnicity and its influence on the gut microbiome of different groups, including African Americans, should be considered in health evaluations and tailored interventions.

To assess whether these findings reflected true compositional differences rather than dispersion effects, the test for homogeneity of multivariate dispersion was conducted, which revealed significant differences for ethnicity (F = 32.537, *p* < 0.0001). This suggests that variability within ethnic groups may contribute to the observed differences. In contrast, dispersion differences for AA status and sex were not significant, indicating that observed community variation within AA and within gender groups is unlikely to be driven by unequal within-group variance. This distinction strengthens the interpretation of the PERMANOVA results specific to AA representation while underscoring the need for more research and a deeper understanding of how gut microbiome varies in underrepresented groups. Overall, these findings contributed to the broader knowledge that microbial composition is shaped by multiple demographic and health factors, with ethnicity emerging as one of the strongest predictors. The influence of ethnicity, including African American status, highlighted the complex determinants of human microbiome structure.

Similarly, although ANCOM-BC corrects for biases due to differences in sampling fractions and accounts for overdispersion in individual taxa [23], it does not explicitly model group-level multivariate dispersion. Our permutation test for homogeneity of dispersion indicated that within-group variability for AA status was not significant, suggesting that differential abundance results are unlikely to be confounded by unequal dispersion across groups. Differential abundance analysis via ANCOM-BC revealed significant findings, highlighting taxa that differ between healthy AA and healthy non-AA groups. The significant increase in butyrate-producing bacteria, including *Clostridium sensu stricto 1*, *Agathobacter*, and *Eubacterium siraeum* group, aligns with previous research highlighting their essential role in intestinal health [34,35]. At the same time, more research is needed to determine which species of *Clostridium sensu stricto* genus are enriched in African Americans since studies have shown some species to be pathogenic in animals and plants [36]. For instance, one clinical study characterizing the fungal gut mycobiota in Current Depressive Episode (CDE) has shown enrichment of *Clostridium sensu stricto 1* with a decrease of *Dialister* [37]. Similarly, a clinical study analyzing patients with IBD has also shown an increased abundance of *Clostridium sensu stricto 1,* indicating its association with upregulation of metabolic pathways of IBD [38]. The decreased abundance of most *Bacteroides* strains is positive because previous findings have demonstrated their enrichment in polyp-positive samples as well as correlation with Alzheimer’s Disease (AD) [39,40]. However, depleted *Bacteroides* cannot be considered completely optimistic either since they have a complex relationship within the gut, playing both a beneficial and harmful role as well [40,41]. Understanding the metabolomic role of the species present can be an avenue of research to elucidate this role. Consistent with these findings, the enrichment of *Agathobacter* and *Eubacterium siraeum* represents a positive outcome, as a previous study has shown its decrease with cognitive impairment and AD [42,43], and *Agathobacter* abundance with better overall survival in multiple myeloma and advanced non-small cell lung cancer [44,45].

The reduction in *Dialister* observed in our cohort is also notable since previous research has linked reduced *Dialister* abundance with depression [37,46,47]. Similarly, *Dialister* is one of the taxa besides *Phascolarctobacterium* that consumes succinate in the gut. During the breakdown of carbohydrates into short-chain fatty acids (SCFAs), succinate is an intermediate metabolite [48]. Depleted *Dialister* can point to lower levels of succinate being metabolized. which could lead to gut dysbiosis. Abnormally increased levels of succinate have been shown in IBD, Crohn’s disease (CD), and ischemia/reperfusion (I/R) [48]. Additionally, the decreased abundance of *Christensenellaceae R-7 group* warrants further investigation, as Brooks and colleagues identified this family as exhibiting significant ethnic-specific variations [11]. These findings collectively suggest that the gut microbiome profile observed in our African American participants may reflect distinctive adaptations that could influence various health outcomes, including metabolic function, cognitive health, and inflammatory status.

The notable enrichment of the *Lachnospiraceae NK4A136 group* is also significant, as its family, Lachnospiraceae, plays a crucial role in SCFA production. This family is consistently associated with various diseases, including diabetes, IBD, CKD, liver disease, obesity, and metabolic syndrome [49]. The observed elevation in *Bifidobacterium* (genus under Actinobacteria) supports findings of higher Actinobacteria phyla presence in Black women compared to White women, regardless of insulin sensitivity [50]. At the same time, one study showed an association of the abundance of *Bifidobacterium* with psychological stress [51]. Another study focusing on Major Depressive Episode (MDE) also showed an increase in *Bifidobacterium* along with the relative abundance of Lachnospiraceae and Bifidobacteriaceae [52]. While *Bifidobacterium* is considered important for gut health as well as immunity, the consistent association of these certain taxa with cognition and mental health warrants further examination [53]. It is also alarming that African Americans’ health may be disproportionately impacted, with microbial influences potentially exacerbating existing socioeconomic disparities.

The limited research on African American gut microbiomes poses a major obstacle to understanding healthcare comprehensively. Past studies have been sporadic and lack depth, failing to reflect the full microbiome diversity within this group. While our dataset contains a comparatively higher proportion of African American samples than most public microbiome datasets, our machine learning findings emphasize this challenge further. The top-performing model (LightGBM) showed a noticeable bias in prediction accuracy among different ethnic groups. It achieved high accuracy for Caucasian subjects but was less effective for minority groups, including African Americans, Hispanic/Latino, Multiracial, and Other categories. Even with the implementation of stratified sampling to reduce this imbalance, the results should be interpreted with this limitation in mind, as this trend indicates that microbiome signatures for Caucasians might be more distinct or better represented in the training data.

This disparity in representation is reflected in our model metrics, explaining the observed gap between the relatively high overall accuracy (76.6%) and the lower macro-averaged precision, recall, and F1 scores on the validation set. The difficulty in accurately classifying underrepresented ethnic groups highlights a critical need for more diverse and representative microbiome datasets that better capture biological variation across all ethnic populations.

However, our feature importance analysis identified key bacterial taxa linked to ethnicity prediction, providing further biological context for the observed classification patterns. The prominence of *Lachnoclostridium* as the top microbial feature matches our differential abundance results, which show increased butyrate-producing bacteria in African American participants. The importance of multiple *Bacteroides* genera is noteworthy, given our finding of decreased abundance of most *Bacteroides* strains in African Americans compared to earlier studies. Additionally, the importance of the *Blautia* genus highlights the potential relevance of butyrate-producing bacteria in ethnic microbiome differences, which may have implications for metabolic health disparities [54,55]. The taxonomic distribution of predictive genera suggests that strain-level variations within the same family contribute differently to ethnicity prediction, revealing microbial signatures that may be missed in broader taxonomic analyses.

Given these patterns, future experimental work should validate microbial function by exploring metabolic pathways of the relevant taxa. The enrichment observed in differential abundance analysis, along with the top features identified by the machine learning models, highlighted genera primarily from butyrate-producing families such as Lachnospiraceae (*Agathobacter*, *Lachnospiraceae NK4A136 group*, *Lachnoclostridium*, *Blautia*), Clostridiaceae (*Clostridium sensu stricto 1*), and Ruminococcaceae (*Eubacterium siraeum*, *UBA1819*, *Subdoligranulum*), which underscores the potential for enhanced SCFA-mediated modulation of host metabolism and neuroinflammation in African Americans [54,55]. Conversely, the depletion of *Dialister*, genus previously associated with depression, suggests possible microbial pathways linked to the gut–brain axis [56]. Similarly, *Bacteroides* and *Alistipes* genera were the remaining predictors in the machine learning model. Experimental studies could prioritize these genera to elucidate their specific metabolic contributions as well. Integrating metagenomic and metabolomic approaches and employing gnotobiotic mouse models colonized with African American-enriched taxa could provide causal insights into how these microbial communities influence neuroinflammatory and metabolic pathways relevant to mental health outcomes [57]. Such studies can bridge the gap between microbiome composition and functional impact, advancing understanding of ethnic-specific microbial contributions to health disparities. Methodologically, the study used advanced computational techniques to analyze microbiome composition, highlighting the potential for more sophisticated approaches to understanding population-specific variations. The findings indicate that future research should prioritize diverse representation, develop standardized methods, and explore the complex relationships between genetics, environment, and microbiome composition in African American populations.

This research is critical to addressing health disparities and developing more personalized, equitable healthcare approaches. By illuminating the unique microbiome characteristics of African Americans, the study opens new avenues for understanding how microbial composition may influence health outcomes, potentially leading to more targeted prevention and treatment strategies that recognize the biological diversity within this population.

## 5. Limitations of the Study

Prior to discussing the broader implications of these findings, it is important to consider the study’s limitations. After collecting datasets across 15 studies, African Americans represented only ~15% of the total dataset, while Caucasians represented ~60%, and the remaining ethnicities together represented ~25%. While the proportion of African American samples in this dataset exceeds that of most publicly available microbiome resources and represents a novel scale of inclusion, the imbalance relative to other ethnic groups may still introduce bias in downstream analyses. For machine learning models that rely on balanced class distributions, stratified sampling was applied to mitigate this effect, but differential representation likely influenced model performance and interpretability across ethnic groups. Similarly, key environmental and lifestyle variables such as diet, socioeconomic status, and medication use were not collected, which limits the ability to fully disentangle biological variation from external confounders and that may also influence diversity metrics. To minimize methodological heterogeneity, datasets generated using the Illumina MiSeq platform and targeting the V4 region of the 16S rRNA gene were prioritized. However, due to the necessity of including studies that enabled ethnicity-based comparisons, some datasets employing alternative sequencing platforms and primer regions were incorporated (Table S1). This inclusion may have introduced variability affecting diversity metrics and differential abundance analyses. Finally, while the observed taxonomic associations are compelling, they remain correlative and should be interpreted cautiously until validated through controlled, longitudinal, and functional studies.

## 6. Conclusions

This research investigation signifies the first comprehensive meta-analysis of the gut microbiome with a specific focus on African Americans and ethnicity as a key feature. It serves as a valuable foundation for future research on underrepresented groups, aiding in the identification of microbial biomarkers that may contribute to health disparities.

Our feature importance analysis revealed taxonomic patterns, especially involving *Lachnoclostridium*, *Bacteroides*, and *Blautia* genera, as well as taxa linked to cognition and mental health, like *Dialister* and *Bifidobacterium*. These findings merit further exploration through experimental studies, longitudinal monitoring, and integration with host genetic data to elucidate functional roles and gene–microbe interactions.

Future work could also explore culturally tailored dietary or intervention studies for African American populations and the development of ethnic-specific microbiome reference ranges to enhance clinical interpretation and precision medicine approaches.

Finally, this study underscores the critical need for greater representation in microbiome research. Building community-engaged research practices and partnerships with historically Black institutions could improve recruitment and retention of African American participants, ultimately leading to a more equitable and comprehensive understanding of human microbiome diversity.

## Figures and Tables

**Table 1:** Prevalence Analysis of Top Phyla with Significant Pairwise Comparisons. The table shows prevalence (%) of the top five bacterial phyla among African American (AA), Asian (A), and Caucasian (C) groups. Coefficients (Coeff. *) indicate effect size from the pairwise comparison, with associated *p*-values (*p* val **). Significant differences were observed, with Firmicutes, Bacteroidota, and Actinobacteriota being more prevalent in Caucasians compared to African Americans, while Proteobacteria and Verrucomicrobiota were more prevalent in Asians compared to Caucasians.

Taxon (Phylum)	AA	C	A	Coeff. *	*p* val **	Comparison
Firmicutes	82.95	99.02	94.78	20.72	9.18705 × 10^−18^	C vs. AA
Bacteroidota	84.09	96.31	90.76	4.93	2.053122 × 10^−8^	C vs. AA
Actinobacteriota	78.41	92.4	87.15	3.34	2.426465 × 10^−7^	C vs. AA
Proteobacteria	80.11	88.93	75.9	0.39	7.817304 × 10^−7^	A vs. C
Verrucomicrobiota	43.75	62	24.1	0.19	6.707755 × 10^−27^	A vs. C

Coeff. *: effect size coefficient; *p* val **: adjusted *p*-value.

**Table 2:** ASV features identified by the ML model and corresponding Taxon relation. ASVs selected as important features by the ML model are listed with their taxonomic assignments at the kingdom, phylum, class, order, family, and genus levels. NA indicates taxa that could not be resolved at a given level.

	Kingdom	Phylum	Class	Order	Family	Genus
ASV1575	Bacteria	Firmicutes	Clostridia	Lachnospirales	Lachnospiraceae	Lachnoclostridium
ASV1451	Bacteria	Bacteroidota	Bacteroidia	Bacteroidales	Bacteroidaceae	Bacteroides
ASV1514	Bacteria	Bacteroidota	Bacteroidia	Bacteroidales	Bacteroidaceae	Bacteroides
ASV1450	Bacteria	Firmicutes	Bacilli	RF39	NA	NA
ASV7189	Bacteria	Bacteroidota	Bacteroidia	Bacteroidales	Bacteroidaceae	Bacteroides
ASV9672	Bacteria	Firmicutes	Clostridia	Lachnospirales	Lachnospiraceae	Blautia
ASV1449	Bacteria	Firmicutes	Clostridia	Lachnospirales	Lachnospiraceae	Blautia
ASV1531	Bacteria	Bacteroidota	Bacteroidia	Bacteroidales	Rikenellaceae	Alistipes
ASV12682	Bacteria	Firmicutes	Clostridia	Oscillospirales	Ruminococcaceae	UBA1819
ASV18151	Bacteria	Bacteroidota	Bacteroidia	Bacteroidales	Bacteroidaceae	Bacteroides
ASV1454	Bacteria	Firmicutes	Clostridia	Oscillospirales	Ruminococcaceae	Subdoligranulum

## Data Availability

Model codes and data are available on GitHub. https://github.com/nush320/Gut-Microbiome (accessed on 10 March 2025).

## Supplementary Materials

The following supporting information can be downloaded at: https://www.mdpi.com/article/10.3390/ijerph22101591/s1, Figure S1. Top 15 Feature Importance for LightGBM; Table S1. Dataset Characteristics; Table S2. Ethnicity and Study Groups breakdown for each Dataset; Table S3. Model Comparison on Validation Set.
