# Supplementary material for: Meta-Analysis of the Gut Microbiome: An African American Representation
Source: Int J Environ Res Public Health. 2025 Oct 20;22(10):1591. doi: 10.3390/ijerph22101591 (PMC12562341; doi:10.3390/ijerph22101591)
Supplement: Supplementary file 1 [file ijerph-22-01591-s001.zip › ijerph-3904938-supplementary.pdf]

Supplementary Materials

Table S1. Dataset Characteristics

Index	Dataset	Disease	H/D	Ethnicity	City	Continent	Instrument	Region
1	Ang et al., 2021	Obesity	0/23	East Asians, Caucasian	San Francisco Bay Area	North America	Illumina MiSeq	V4
2	Bao et al., 2021	Food Allergic	13/23	Caucasian, Asian, Hispanic, Black, Asian	California cities - Palo Alto, Mountain View,	North America	Illumina MiSeq	V4
3	Barengolts et al., 2018	Obesity, T2D, Psychiatric disorders	0/99	AA	Chicago, IL	North America	Illumina MiSeq	V3-V4
4	Baxter et al., 2016	Adenoma sizes including Cancer	172/318	Multi-ethnic cohort	Toronto, Boston, Houston, Ann Arbor	North America	Illumina MiSeq	V4
5	Brim et al., 2013	Colon Polyps vs Non	6/6	AA	Washington DC	North America	454 GS FLX Titanium	V1-V3
6	Dadkhah et al., 2019	Colon Polyps vs Non	236/316	Multi-ethnic cohort	Washington DC	North America	Ion Torrent PGM	V1-V2
7	Jacobs et al., 2023	IBS	174/312	Multi-ethnic cohort	Los Angeles and Buffalo	North America	Illumina HiSeq 2500	V4
8	McLeod et al., 2023a	Obese and MCI	0/66	AA, Mixed, Other	Chicago, IL	North America	Illumina MiSeq	V4
9	Piawah et al., 2023	CRC	0/30	Multi-ethnic cohort	San Francisco	North America	Illumina MiSeq	V4
10	Saffouri et al., 2019	NA	38/0	Caucasian, Other	Minnesota	North America	Illumina MiSeq	V4
11	Vandoni et al., 2023	AM	0/31	Caucasian	Milan, Italy	Europe	Illumina MiSeq	V3-V4
12	Yu et al., 2022	RA	26/26	Chinese	Taizhou	Asia	Illumina NovaSeq 6000	V3-V4
13	Dhakan et al., 2019	NA	110/0	Indian	Bhopal and Kerala	Asia	NextSeq 500	V3
14	Huang et al., 2019	NA	13/0	Chinese	Shanghai	Asia	Illumina MiSeq	V4-V5

Table S2. Ethnicity and Study Groups breakdown for each Dataset

233333	Study Group	City	AA	C	A	H/L	N	M	O	Total
Ang et al., 2021	Healthy	San Francisco	0	12	13	0	0	0	0	25
	Obese		0	13	10	0	0	0	0	23
Bao et al., 2021	Healthy	San Francisco	0	7	3	1	0	1	0	12
	Allergic		2	15	3	1	0	1	0	22
Barengolts et al., 2018	Healthy	Chicago	46	0	0	0	0	0	0	46
	T2D		49	0	0	0	0	0	0	49
	NA		1	0	0	0	0	0	0	1
Baxter et al., 2016	Healthy	Ann Arbor	3	48	0	0	0	0	0	51
		Boston	3	34	0	0	0	0	1	38
		Houston	2	54	14	0	0	0	3	73
		Toronto	0	25	0	0	0	0	0	25
	adenoma	Ann Arbor	3	32	1	0	0	0	0	36
		Boston	5	32	0	0	0	0	0	37
		Houston	1	8	0	0	0	0	0	9
		Toronto	2	135	5	0	0	0	1	143
	cancer	Ann Arbor	0	26	0	0	0	0	0	26
		Boston	0	56	0	0	0	0	0	56
		Houston	2	20	1	0	0	0	1	24
		Toronto	0	17	1	0	1	0	0	19
Brim et al., 2013	Healthy	DC	6	0	0	0	0	0	0	6
	Polyp		6	0	0	0	0	0	0	6
Dadkhah et al., 2019	Healthy	DC	6	62	2	0	0	0	0	70
	Polyp		8	93	0	0	0	0	0	101
Jacobs et al., 2023	Healthy	Los Angeles	9	21	15	25	0	6	0	76
	IBS		22	112	29	46	1	17	0	227
		Buffalo	3	42	0	2	0	2	0	49
McLeod et al., 2023a	Obese & MCI	Chicago	62	0	0	3	0	0	1	66
Piawah et al., 2023	CRC	San Francisco	4	7	0	7	0	0	0	18
Saffouri et al., 2019	Healthy	Minnesota	0	23	0	0	0	0	2	25
Vandoni et al., 2023	AM	Milan	0	31	0	0	0	0	0	31
Yu et al., 2022	Healthy	Taizhou	0	0	26	0	0	0	0	26
	RA		0	0	26	0	0	0	0	26
Dhakan et al., 2019	Healthy	Bhopal	0	0	49	0	0	0	0	49
		Kasaragod	0	0	39	0	0	0	0	39
Huang et al., 2019	Healthy	Shanghai	0	0	13	0	0	0	0	13
		**Total**	245	925	250	85	2	27	9	1543

Table S3. Model Comparison on Validation Set

	accuracy	precision_macro	recall_macro	f1_macro	roc_auc
**LightGBM**	0.766234	0.454133	0.386356	0.405635	0.835936
**XGBoost**	0.744589	0.445580	0.383134	0.403960	0.825866
**AdaBoost**	0.649351	0.430297	0.265266	0.281289	0.781372
**RandomForest**	0.649351	0.438636	0.216216	0.211374	0.828995

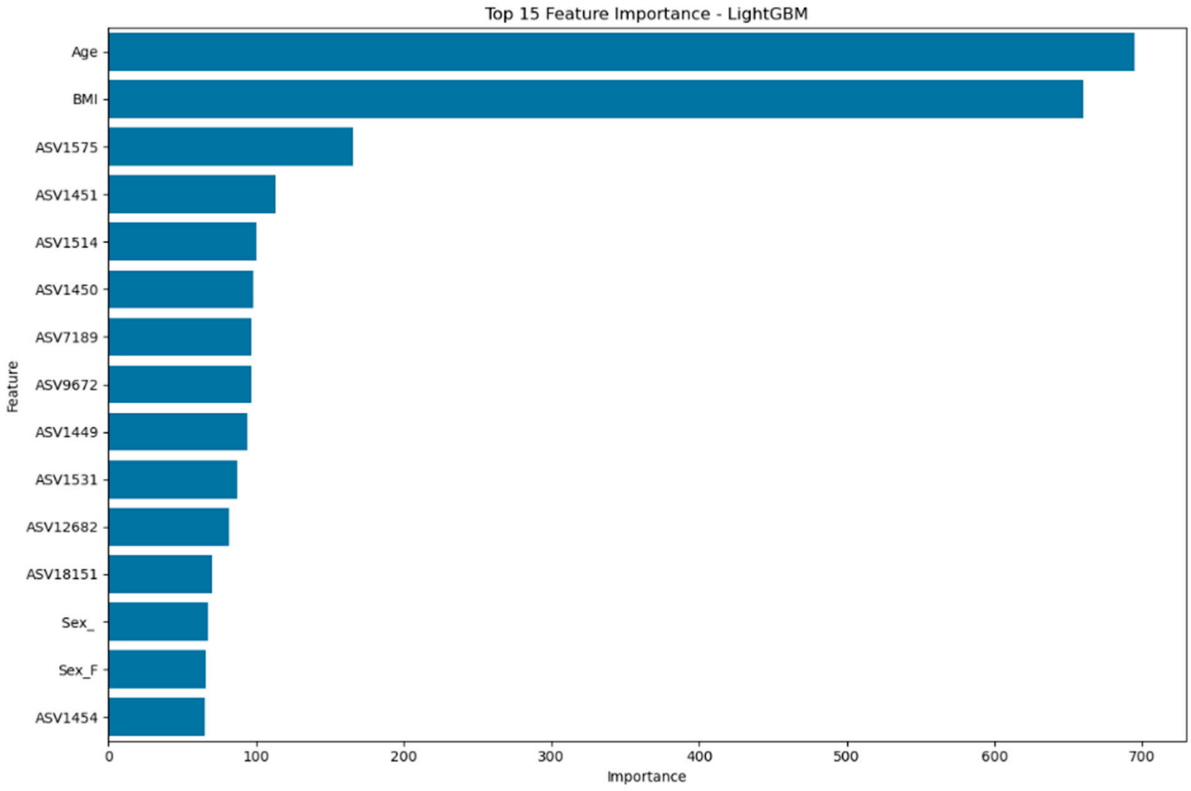

Figure S1. Top 15 Feature Importance for LightGBM
